# Co-designing implementation strategies for the WALK-Cph intervention in Denmark aimed at increasing mobility in acutely hospitalized older patients: a qualitative analysis of selected strategies and their justifications

**DOI:** 10.1186/s12913-021-07395-z

**Published:** 2022-01-02

**Authors:** Jeanette Wassar Kirk, Per Nilsen, Ove Andersen, Byron J. Powell, Tine Tjørnhøj-Thomsen, Thomas Bandholm, Mette Merete Pedersen

**Affiliations:** 1 Department of Clinical Research, Copenhagen University Hospital, Amager and Hvidovre, Hvidovre, Denmark; 2 Department of Public Health, Nursing, Aarhus University, Aarhus, Denmark; 3 Department of Health, Medical and Caring Sciences, Linköping University, Linköping, Sweden; 4 Brown School, Washington University in St. Louis, St. Louis, MO USA; 5 Department of Health and Social Context, National Institute of Public Health, University of Southern Denmark, Copenhagen, Denmark; 6 Department of Orthopedic Surgery, Physical Medicine & Rehabilitation Research-Copenhagen (PMR-C), Copenhagen University Hospital, Amager and Hvidovre, Hvidovre, Denmark; 7 Department of Physical and Occupational Therapy, Physical Medicine & Rehabilitation Research-Copenhagen (PMR-C), Copenhagen University Hospital, Amager and Hvidovre, Hvidovre, Denmark

## Abstract

**Background:**

Selecting appropriate strategies to target barriers to implementing interventions represents a considerable challenge in implementation research and practice. The aim was to investigate what categories of implementation strategies were selected by health care practitioners and their managers in a co-design process and how they justified these strategies aimed at facilitating the implementation of the WALK-Cph intervention.

**Methods:**

The study used a qualitative research design to explore what implementation strategies were selected and the justifications for selecting these strategies. Workshops were used because this qualitative method is particularly well suited for studying co-design processes that involve substantial attention to social interaction and the context. Data were 1) analyzed deductively based on the Proctor et al. taxonomy of implementation strategies, 2) categorized in accordance with the ERIC compilation of implementation strategies by Powell et al., and 3) analyzed to examine the justification for the selected strategies by the Proctor et al. framework for justifications of implementation strategies.

**Results:**

Thirteen different types of implementation strategies were chosen across two hospitals. The deductive analysis showed that selection of implementation strategies was based on pragmatic and theoretical justifications. The contents of the two types of justifications were thematized into nine subthemes.

**Conclusion:**

This study contributes with knowledge about categories and justification of implementation strategies selected in a co-design process. In this study, implementation strategies were selected through pragmatic and theoretical justifications. This points to a challenge in balancing strategies based on practice-based and research-based knowledge and thereby selection of strategies with or without proven effectiveness.

**Supplementary Information:**

The online version contains supplementary material available at 10.1186/s12913-021-07395-z.

## Background

Implementation strategies constitute the “how to” of getting evidence-based interventions (EBIs) into practice [1–4]. Powell et al. [5] define implementation strategies as “methods or techniques used to enhance the adoption, implementation, sustainment and scale-up” of interventions. Strategies can vary in complexity from single to multifaceted [3, 5]. Selecting appropriate strategies to target barriers to implementing interventions represents a considerable challenge in implementation research and practice [4–9], e.g., choosing appropriate strategies to influence poor motivation or negative attitudes concerning the use of a new intervention. Systematic approaches are needed to design and tailor implementation strategies that integrate evidence, theory, and stakeholder perspectives [7, 10, 11]. Theories, models, and frameworks have been developed to facilitate the matching of relevant strategies to specific barriers [4, 6, 8–10, 12]. However, the use of inconsistent terminology and inadequate descriptions of strategies [3, 13] make it difficult to identify optimal strategies and to advance our understanding of how and when different implementation strategies are effective [14–16]. Implementation strategies tend to be entangled in the context, which can affect the effectiveness of the strategies [17]. Thus, if the identified barrier is insufficient skills training can have more impact on health care practitioners’ performance than a “default” strategy, which has always been selected in the past regardless of what barriers might have existed.

Developing and selecting implementation strategies can be achieved in different ways, e.g., through expert panels [18] that involve both implementation researchers and clinical experts or through stakeholder involvement in co-design processes to achieve more contextually adapted strategies to increase the likelihood of successful implementation of EBIs [19–21]. Co-design has been defined as “the creativity of designers and people not trained in design working together in the design development process” [22]. Co-design is used to develop solutions to complex problems [23] and is intended to be a social and democratic process [24]. Several advantages of accounting for stakeholder priorities in co-design processes have been described, including improved credibility of the results and optimization of the implementation of EBIs through better understanding of the intervention-context fit [7, 11, 25–27]. Some studies have investigated the use of co-design processes involving practitioners (i.e., non-researchers) together with researchers to develop and select implementation strategies for implementation of EBIs in health care [28–30].

This study was part of the WALK-Copenhagen (WALK-Cph) project [31], which was initiated to implement a multi-facetted intervention to increase mobility in older patients acutely admitted to two medical departments at two university hospitals in Denmark (Hospital X and Hospital Y). The project used a Hybrid II design, with a dual focus on the development and the implementation of the intervention [32]. This study report results from study 1b, 2d and 2 h in relation to the overall WALK-Cph project (Additional file 1: Appendix S1). The intervention was co-designed in a collaborative process between researchers, one design architect employed at the hospital, health care practitioners, patients, and their relatives. The co-design of the strategies to implement the intervention involved only researchers and health care practitioners, who were to be responsible for implementing the intervention. The content of the intervention is shown in Additional file 2: Appendix S2. In response to lack of empirical knowledge on the involvement of health care practitioners in co-designing implementation strategies, the aim of this study was to investigate what implementation strategies were selected by health care practitioners and their managers and how they justified these strategies aimed at facilitating the implementation of the WALK-Cph intervention.

## Methods

### Study design

The study used a qualitative research design, interactive workshops, which is particularly well suited for studying co-design processes that involve substantial attention to social interaction and the context [22]. The study is reported using the Standard for Reporting Implementation Studies (StaRI) checklist [33].

### Study setting

The WALK-Cph project was carried out in Denmark. The health care system is funded by taxes and provides free treatment for all citizens for primary medical care, hospital care, and home-based care services. The project involved two hospitals (Hospital X and Hospital Y) represented by: Department X, a Department of Endocrinology and a Department of Occupational and Physical Therapy and the associated municipality (Municipality X), and Department Y, a Department of General Medicine including occupational therapists and physiotherapists and the associated municipality (Municipality Y). The departments were located at two public university hospitals in the Capital Region of Denmark and were similar in size and staff composition (Additional file 3: Appendix S3). The municipalities, in which the hospitals are located, were also part of the project since they were recipients of patients discharged from the two intervention departments. The municipalities were represented by their rehabilitation units and the home care services. Thereby, in daily practice there was a close collaboration between the two medical departments and the two municipalities. Before designing the implementation plan, all stakeholders (staff from Hospital X and Y, the municipalities, patients and relatives) designed the interventions together (Kirk et al. in review).

### Co-design workshops

**Fig. 1:**
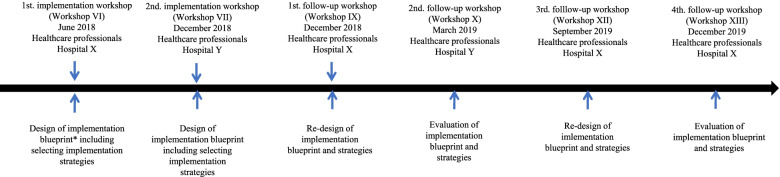
Process of initial and follow-up co-design workshops

The stakeholders worked together in two initial co-design workshops held in June 2018 with Hospital X participating in the 1st workshop and in December 2018 with Hospital Y participating in the 2nd workshop to develop the implementation plans (Fig. 1, Workshops VI + VII). The workshops were followed-up by four co-design workshops (Fig. 1, Workshops X-XIII), with the purpose to adapt the implementation plan. The workshops were inspired by Pavelin et al. [34], who defined interactive workshops as “a structured set of facilitated activities for groups of participants who work together to explore a problem and its solutions, over a specific period of time, in one location.” The co-design workshops were designed to encourage creativity and production of ideas among the participants for the proposed implementation process, e.g. by using coloured sticky notes and through the use of a Conjoint Analysis (CA) [35] to prioritize potential implementation strategies suggested by the stakeholders. Both sticky notes and CAs can function as mediating tools to generate ideas and thus increase the likelihood of implementation of an intervention. The first author (JWK) and the other project manager (MMP) had the overall responsibility for leading and facilitating the co-design process of the workshops (Fig. 1). Neither JWK nor MMP has a formal education in design, but JWK has experience with user-involvement, co-creation methods, facilitation and process competencies [36]. The hospital design architect, who participated in the design of the intervention, had a formal education in co-creation.

In this study, facilitation is defined as helping a group to achieve a common goal and assist them in achieving the desired results. This is done without taking a stand or being prescriptive, but by focusing on the dialogue and context of the discussions [37]. Facilitation basically consists of two components: design of the process and the facilitation itself [37]. The design of the process is described below in form of involving stakeholders and the structure of the workshops.

In the facilitation situation, the facilitators functioned as neutral catalysts and ensured that all relevant perspectives were accounted for. For instance, the facilitators asked about the same issues across groups and observed and tried to interpret body language that signaled a positive or negative attitude. The dialogue was guided through open and simple questions and by taking responsibility for the energy in the workshop. This was achieved by means of alternating between common dialogue and allowing time for reflection and pauses [38].

The stakeholders were health care practitioners and frontline managers from the two intervention hospitals. From Hospital X they were one nurse, one ward physician, one frontline manager (nurse), three physiotherapists and two head managers (physiotherapists). From Hospital Y they were one nurse, one frontline manager (nurse), two physiotherapists, one frontline manager (physiotherapist) and one occupational therapist (Table 1). In Denmark, the frontline managers generally have expertise in quality improvement and have the daily responsibility for patient care and contact with the staff. They are usually responsible for implementing new interventions and monitoring the quality of existing processes. Head managers have the overall responsibility for implementing interventions. The research team participating in the workshops consisted of a professor, a postdoc researcher, and a research assistant with a background in physiotherapy, and a PhD student and a postdoc researcher with a nursing background.

**Table 1:** Participating health care practitioners

	Profession (***N*** = 15)	Years of experience	Example of groups for workshops	Workshops
**Hospital X**			Group 1	1st initial, 1st follow-up, 3rd follow-up and 4th follow-up
Endocrinology	Nurse	> 10		
	Frontline manager (nurse)	> 10	Group 1	
	Physician	> 10	Group 1	
Physiotherapy	Frontline manager	> 10	Group 2	
	Head manager	> 10	Group 2	
	Physiotherapist	> 10	Group 2	
Municipality	Physiotherapist	> 5	Group 3	
	Physiotherapist	> 5	Group 3	
	Head manager	> 10	Group 3	
**Hospital Y**				
General Medicine	Nurse	> 10	Group 4	2nd initial and 2nd follow-up
	Frontline manager (nurse)	> 5	Group 4	
	Physiotherapist	> 5	Group 4	
	Occupational therapist	> 5	Group 4	
	Frontline manager (physiotherapist)	> 10	Group 4	
Municipality	Physiotherapist	> 10	Group 4	

Home care service staff only participaated in the initial intervention design work-shop due to ongoing major organizational changes and are therefore not counted in the participants

Before the study started, JWK and a colleague held individual meetings with the head and frontline managers to discuss their department’s capacity regarding who and how many of their health care practitioners they could involve as implementation champions [39] (e.g., someone who is dedicated to promote the implementation of an intervention and thereby overcome resistance that the intervention may create in an organization) [39]. The researchers aimed for variation in inclusion of participants as the managers had knowledge and experience about who might be expected to have positive perceptions of the intervention and who would be more skeptical. This type of knowledge can be useful when implementing new initiatives [40]. In total, 15 people participated in the workshops (Table 1).

The two initial workshops took place in June 2018 (Department X) and December 2018 (Department Y) (Fig. 1). Each workshop lasted 2.5 h, and they were held in meeting rooms at the hospitals. The workshops were co-design sessions in which the health care practitioners (i.e., managers and implementation champions), developed the formal implementation plan (i.e., what strategies, when to perform the strategies, how to use the strategies, and the time required [39]). These workshops were followed by four follow-up workshops with the participants in December 2018 (Department X) and March (Department Y), September (Department X), and December 2019 (Department X). All stakeholders from Hospital X participated in the 1st, 3rd and 4th follow-up workshops. Stakeholders from Hospital Y participated in the 2nd follow-up workshop (Table 1). Only one follow-up workshop was completed with stakeholders from hospital Y because the department was scheduled to close down at the end of the year due to political reforms. The follow-up workshops aimed at refining the implementation strategies (Fig. 1) based on experience from practice and on testing the relevance and appropriateness of the chosen strategies.

The initial workshops were structured as follows: (1) group discussions about how to ensure capacity in relation to implementation (e.g., staff and finances); (2) discussions within and between the groups about perceived implementation barriers; (3) feedback loops whereby the research team presented barriers (Table 2) identified in an earlier ethnographic field study performed in the same setting. Three new barriers emerged in the barrier discussions: continuous management, information exchange and concern about lack of resources (barriers 8–10 in Table 2) [41]; (4) PowerPoint presentation of implementation strategies based on the ERIC compilation [39] and hand-out of written examples where several of the strategies were clearly described; (5) selection and tailoring of implementation strategies from the ERIC compilation to address barriers and define outcome targets of the implementation strategies; (6) a conjoint analysis of identified and prioritized barriers based on Farley et al. [35]; and (7) development of the final implementation plan [39]. The ERIC compilation is a well-established and widely applied taxonomy by Powell [13] that identifies no less than 68 implementation strategies, such as distributing educational materials to healthcare professionals. The use of the ERIC compilation was a way to visualize and make different types of implementation strategies apparent for the participants.

**Table 2:** Description of the barriers [40]

Category	Number	Barrier	Why the barrier occurs?
Professional role	1	Nurses downgrade mobilization/mobility to other tasks	Nurses do not perceive mobilization/mobility as part of the treatment and as part of their core tasks
	2	Nurses and nursing assistants serve self-reliant patients	It is more convenient, and it is a habit
	3	Physiotherapists do not believe that it is their responsibility or task to mobilize patients unless the patients need training	Therapists, nurses, nursing assistants, and physicians gave different meanings to the concept of mobilization/mobility and to the tasks and actions that can be considered mobilization/mobility
	4	The physicians’ lack of involvement in the project	When physicians focused on mobilization/mobility and exercise it was usually in connection with discharge or transfer to other departments
	5	Some physicians’ attitude toward the project and their responsibility to prescribe walk plans	Time pressure and the perception that mobilization/mobility is not part of their core tasks
	6	Physicians are critical in their collaboration with physiotherapists, who they think are working in a parallel world	The physicians seem to think that the therapists take too little responsibility in mobilizing the patients
Organizational	7	Different professions have different rhythms of work, which affects whether the patients get out of their bed	Different rhythms of work and different types of interruptions that create break-ups in the temporality of work affected whether mobilization/mobility succeeded or not
	8	Continuous management support and demand for continuation of both the intervention and implementation of the intervention	Some of the implementation champions experienced that they were constantly presented with new initiatives to be implemented. Therefore, focus from the managers was sometimes lacking, as many actions required management attention
Across health care sectors	9	Information exchange between the hospital and the municipality	Empirical experience shows that there are often problems with citizens’ electronic journals when they are transferred between the two health care sectors. The consequence is that the municipality is not notified if the citizens have received a walk plan in the hospital
Financial	10	Concern about lack of resources (time and staff) to implement the intervention	The implementation of the intervention demands the presence of more physical therapists in the Endocrinology Department, which can be a resource problem

Barriers 1–7 were described previously but were considered central to the implementation for the managers and the implementation champions. Barriers 8–10 emerged at the workshops

The co-design process itself made use of different implementation strategies, e.g. Develop an implementation glossary and Facilitation. These strategies mediated the overall progress of the project and supported the methodological design, including user involvement and co-design. An example of this was the strategy to organize implementation team meetings, which were operationalized as follow-up workshops where the research team continuously provided feedback on analyzed data via the strategy of Audit and feedback. In addition, the strategies were used as examples for the participants, enabling them to see how different types of implementation strategies could be used.

Participants from Hospital X were grouped in three groups within their own department or municipality, while participants from Hospital Y were in one group as there was only one participant from the municipality. By presenting data from the earlier ethnographic field study [41] and barrier screening [42], the assumption was that this would create transparency in the work, create trust between the research team and the participants, and support behavioral changes of the participants in the implementation of the intervention. After the workshops, all participants returned to their departments and municipalities, and they had 2 weeks to complete the final implementation plan, which was then sent to the research team.

The research team met with the health care practitioners four times in follow-up workshops, which lasted approximately 1.5 h each (Fig. 1). These were more unstructured workshops as the participants were placed randomly in a large group. The purpose was to discuss the participants’ experiences with the use of the implementation plan. I.e. what worked or did not work and why. The researchers wrote barriers, facilitators and adaptations of the implementation plan on a whiteboard, and before the end of the workshops, the participants adapted the implementation plan in collaboration. Again, the assumption was that group negotiation about implementation plan adjustments would create transparency and a joint ownership, which was assumed to be a prerequisite for a successful implementation.

### Data collection

Data were collected the same way at all workshops and follow-up workshops. We divided the participants into small groups belonging to the same department (e.g., the municipality). JWK acted as a facilitator during the workshops and ensured that the same topics were discussed in all groups. The facilitator did not contribute with ideas but encouraged input from all participants. The researchers worked on the premise that participants could negotiate appropriate proposals themselves.

All groups were in the same room, which made it difficult to audiotape conversations all the time. Additionally, research team members took notes on: (1) interactions between members within and between groups; (2) justifications for the selected implementation strategies; and (3) contradictions between members within and between groups. All follow-up workshops were audiotaped because they were held in separate rooms in the two hospitals. In total, 8 h of conversations were captured on recordings, 30 pages of notes and 107 pages of transcribed data material were produced.

### Data analysis

First, the first author (JWK) performed a deductive thematic analysis by reading the empirical material line-by-line. Then the meaning units was coded in three steps [43] using different frameworks [3, 13]. The frameworks were used to ascertain consistent labeling and descriptions as well as to provide a theoretical justification for the findings. First, the content of the stakeholders’ proposed implementation strategies was categorized according to the framework of Proctor et al. [3] which emphasizes naming, defining and operationalizing implementation strategies. The operationalization encompasses the specification of the following dimensions: actors, actions, action target (or mechanism through which the strategy works), temporality, dose, and outcome affected. Second, the strategies were categorized in accordance with a taxonomy of strategies by Powell et al. [13]: planning, e.g. helping stakeholders gather data or select strategies, educating, e.g. presentation of strategies of various levels of intensity that can be used to inform stakeholders about the intervention and/or its implementation, financing, restructuring, managing quality, and attending to the policy context. Third, data were analyzed to examine the justifications for the selected implementation strategies by JWK reading and re-reading the field notes and the transcribed material to gain a deeper understanding of the underlying motives for the selected strategies. This data analysis was informed by Proctor et al.’s [3] distinction between empirical, pragmatic, and theoretical justifications. Empirical justifications are based on either research evidence or on individuals’ experience with the evidence-based strategies [44]. Pragmatic justifications specify a clear rationale about what factors need to be addressed and how selected strategies may address them, but do not provide an empirical or theoretical justification for the strategies [45]. Pragmatic justification is based on knowledge derived from “real-world” practice and on “practice-based evidence” [43, 46, 47] derived from the experience of the participants. Theoretical justifications are based on theoretical knowledge and rely on insights gained from a body of knowledge accumulated in a research field or concerning a specific subject [47, 48].

The initial sub-themes for the justification of the implementation strategies were discussed with the research team before the condensation of the final themes. One issue discussed was to secure consensus understandings of the three categories, i.e. empirical, pragmatic and theoretical justifications.

## Results

Thirteen implementation strategies were selected across Hospitals X and Y (Table 3 and Additional file 4: Appendix S4). In Hospital X, all 13 strategies were selected while Hospital Y, selected seven of the 13 strategies. The strategy “change physical structure and equipment” was not associated with a specific barrier but was based on previous experiences of all managers who emphasized the importance of having the equipment ready before the start of the project because this could otherwise become a barrier. Detailed information about each implementation strategy is presented in S4.

**Table 3:** Implementation strategies selected

Number	Name of the strategy
1.	Change physical structure and equipment
2.	Conduct local consensus discussions^a^
3.	Develop educational materials^a^
4.	Information on board meetings with all the staff^a^
5.	Information at physician conferences
6.	Tailor strategies^a^
7.	Identify early adopters
8.	Mandate change
9.	Conduct ongoing training^a^
10.	Reminder^a^
11.	Build a coalition
12.	Audit and feedback^a^
13.	Information meeting with head managers

^a^Strategies selected in both Hospital X and Y

### Categorization of the selected strategies

The results from the deductive analysis of the characteristics of the selected strategies are shown in Table 3 and a short summery can be found in Additional file 5: Appendix S5. Most of the selected strategies could be mapped onto the planning and educating categories in the ERIC compilation of implementation strategies [13]. For example, participants from Hospital X selected the strategy ‘Identify early adopters’, whereas participants from Hospital Y chose the strategy ‘Develop educational materials’ (Table 4). Educating involve different strategies of various levels of intensity that can be used to inform stakeholders about the intervention and/or its implementation. No strategy was selected in the category “attend to policy context” because there was a lack of strategies targeting the institutional level (see the action targets in Table 4).

**Table 4:** Selected implementation strategies into the ERIC compilation of implementation strategies [13]

Planning	Educating	Financing	Restructuring	Managing quality	Attending to the policy context
Tailor strategies	Conduct local^b^ consensus discussions	Provide resources for training^a^	Change physical structure and equipment	Reminder	
Mandate change	Develop educational materials		[Reexamine the implementation]	Audit and feedback	
Build a coalition	Information on board meetings with all the staff		[Facilitation]	Information meeting with head managers	
Identify early adopters	Information at physician conferences			[Organize implementation team meetings]	
	Conduct ongoing training			[Audit and feedback]	
[Conduct local needs assessment]^b^					
[Develop an implementation glossary]					
[Develop a formal implementation blueprint]					
[Conduct a barrier screening]					

^a^This strategy was not discussed but was implicitly decided when the education strategy was chosen

^b^Strategies in brackets were used as part of the co-design itself

^c^Strategy chosen of all departments and munipalities at both Hospitals

### Justifications for the selected strategies

The deductive analysis of the data concerning the rationale for the selected strategies showed that selection was based on pragmatic and theoretical justifications. The contents of the two types of justifications were thematized into nine subthemes (Table 5).

**Table 5:** Themes and subthemes

Themes	Subthemes
Pragmatic justifications (practice-based evidence)	Experience
	Intentions (what is said versus what is done)
	Habits
	Resource limitations
	Sense of shared responsibility
	*Unfamiliar with systematic work with implementation*
Theoretical justifications	Knowledge from organizational theory
	Knowledge from quality improvement
	Profession-related knowledge

#### Empirical justifications

The analysis showed that empirical justifications were not used to select implementation strategies. The participants had various practice-based experiences using some of the evidence-based strategies (e.g., audit and feedback). However, they were not concerned with whether these strategies were evidence-based or not, despite the fact that the research team described some of the strategies and told the participants about the degree to which the strategies were based on evidence.

#### Pragmatic justifications

Four types of pragmatic justifications were identified in the data: experience (knowledge gained through the participants’ own experiences in their professional practice); intentions (the participants’ goodwill to implement the intervention); habits (actions that were more or less automatically enacted, executed without much conscious or deliberate effort); and resource limitations (based on perceived lack of time and/or staff). Two additional types of pragmatic justifications were identified: a sense of shared responsibility for implementing the intervention that developed among the participants and unfamiliarity with systematic implementation work. Neither of these are a part of the taxonomy by Powell et al. [39] or Proctor et al. [3] and neither relied on experience.

#### Experience

The participants often explained how they had good experience from their clinical practice. They did not use the word strategy but talked about “actions” or “plans.” A frontline manager expressed, when addressing the strategy on local consensus discussions:

“We need to start a discussion in the staff group about the importance of getting patients out of bed and the importance of the intervention. Normally it helps to get the intervention implemented.” (Hospital X, notes).

A frontline manager argued when discussing the strategy on identifying early adopters and how and why certain staff should be informed before other staff:

“As soon as possible we need to talk to xx. She is always so positive and open to change. I have a good feeling about her, and I have good experiences with getting her involved.” (Hospital X, notes)

In these discussions, the participants did not relate their justifications to any form of evidence, evaluation or measure, but simply referred to everyday experiences that had become useful and significant to them in their daily practice.

#### Intentions

A second category of pragmatic justification was selecting strategies based on intentions. These intentions were also grounded in experience, but were more hypothetical and/or normative, i.e., expressed in terms of what they think they “should do” or would be “best to do.” An implementation champion said:

“I think it is best if you [addressing the frontline manager] talk about the importance of implementing this intervention every day at board meetings. Or, maybe I can do it ?[pause] On the other hand, you have the mandate and they [the staff] listen to you.” (Hospital X, transcribed notes)

This statement indicates that the strategy on information on board meetings with all the staff was justified based on an idea that the implementation champion could perform this task even if it was new to her. The intentional justification was grounded in power and position based on experience, where the frontline manager and the implementation champions decided that the strategy should be carried out by a person with a mandate to ensure that the strategy is implemented (Table 3). This also became evident at the follow-up workshop, where it emerged that it had not been possible to implement the strategy due to oversight and lack of manager presence at the board meetings.

#### Habits

Some strategies were selected with the justifications that the staff were used to employing the strategies, which were more or less automatically enacted, i.e. a form of habit-based justification. An implementation champion described:

“We normally develop written material. I know I have to deliver the material to the patients and students, but I often forget to do it” (Hospital Y, notes)

Written material is an educational implementation strategy [39] often intended to increase knowledge. Despite the awareness that the participants often forgot to hand out written material and the fact that no barrier concerning lack of knowledge was identified, this was chosen as a strategy (Tables 2 and 4).

Similarly, the strategy of conducting ongoing training was addressed by the physiotherapist implementation champions and managers as something they always did, although no barrier indicated that the staff lacked relevant skills (Additional file 4: Appendix S4).

At a follow-up workshop, it became clear for several frontline managers and implementation champions that they normally used implementation strategies at the outset of projects but did not employ them throughout the process. A frontline manager expressed:

“We are always so motivated in the beginning of a new innovation, but the hard part is to ensure focus along the way. I have learned that it is just as important to use implementation strategies along the way in the projects as at the start if we are to ensure that the interventions are implemented.” (Hospital X, transcribed notes)

The participants described how high motivation at the beginning of a project led to a focus on the use of implementation strategies. When the motivation decreased over time, the focus on implementation strategies declined, resulting in limited use of the strategies. The strong motivation at the outset of the project appeared to be a justification for predominantly selecting strategies in the category of ‘planning’ (Table 4).

#### Resource limitations

Resource limitations in the form of time and/or staff restrictions provided another pragmatic justification. As part of the intervention, the physiotherapists and the nurses should collaborate on deciding what level of walking plan should be delivered to the patients. For the therapists in X, it required new ways of collaborating and allocation of more time to get the intervention implemented. As a frontline manager said:

“It is not possible for me to allocate more time for my therapists in the departments. If the implementation of the intervention requires more resources, then I think financial resources must be dedicated so that I can free up time for my therapists.” (Hospital X, transcribed notes)

The quote reflected resource limitations, which could justify the selection of a financing strategy, such as ‘Access new or existing money to facilitate the implementation’. Resources were not discussed as a strategy in Y because the therapists were affiliated to the department. The way of organizing seemed important for selection and justification of implementation strategies. Despite discussions about resources, no financial implementation strategies were selected, as shown in Table 4.

In general, these four categories of pragmatic justifications were characterized by a perception of importance for the participants as well as on intentions, habits, and resource limitations rather than on scientific evidence or systematic evaluations of what research has established works and what does not work. Two additional categories of pragmatic justifications were identified, but neither of these relied on any specified experience.

#### Sense of shared responsibility

The discussions in the workshops showed that the participants’ knowledge about each other’s work within and across health care sectors was limited, even though the municipalities and both medical departments had worked together for many years. They talked about how they had experiences with complicated collaborations across health care sectors in relation to implementing interventions (e.g., communication). They knew that there was not a one size fits all strategy to overcome these complications. During the discussions, one of the implementation champions suggested a visit across sectors as part of the preparation for the implementation of the intervention:

“It seems a good idea to visit each other before we start to test the intervention. I think it is important because it gives a better understanding and acceptance of how we work with the patients, also if there are problems getting it [the intervention] implemented.” (Hospital X, transcribed notes)

A head manager followed up:

“It also helps create responsibility between us, so everyone does their best getting the intervention implemented.” (Hospital X, notes)

None of the participants disagreed even though no one had previously taken the initiative to visit across health care sectors. The participants visited each other, which contributed to the strategy of building a coalition between the departments and the municipalities to create a sense of shared responsibility for the implementation of the intervention.

#### Unfamiliar with systematic work with implementation

Throughout the process of developing the implementation plan and selecting strategies to overcome barriers, it appeared that none of the participants had previously worked with implementation strategies and plans in a systematic way. A frontline manager commented:

“In my daily practice, I normally implement a lot of new innovations, big or small innovations. But I have never known many of the 'tools'[implementation strategies] that I have been introduced to in this process. It's actually scary.” (Hospital X, transcribed notes)

This statement was followed up by an observation by an implementation champion:

“It is interesting that implementation is a mandatory part of my job, but I have never received any education in implementation.” (Hospital X, notes)

In general, the importance of creating a shared responsibility between the participants led to the development of a new implementation strategy of a visit across sectors. This shared responsibility could have consequences for the trust or distrust between the participants, depending on the outcome of the implementation. Few participants had implementation science knowledge, but by participating in the project, they learned about the importance of working with implementation strategies in a systematic manner, which contributed to the selection of strategies on, for instance, conducting barrier screening and tailoring strategies. The sparse knowledge of implementation research also became apparent in the theoretical justifications.

#### Theoretical justifications

Three types of theoretical justifications were identified. These justifications were based on theoretical knowledge derived from three areas of research: quality improvement (QI), organizational theory, and professional knowledge.

#### Knowledge from quality improvement

QI knowledge was the basis for selecting the strategy of *audit and feedback*. Head and frontline managers are responsible for the quality in their departments and are acquainted with QI knowledge, which they referred to. For example, a frontline manager stated:

“I know a little about quality improvement, the PDSA circle or audit and feedback.”

#### Knowledge from organizational theory

In contrast to the frontline managers, both head managers selected strategies with a knowledge basis in organizational theory concerning implementation rather than QI knowledge. They had acquired this knowledge through other education. A head manager said:

“As part of my Master’s in public management, I have learned about implementation. I know the importance of creating an understanding of the importance of change through motivation. Try to create a positive burning platform [a difficult situation which it is crucial to change].” (Hospital X, transcribed notes)

The theoretical knowledge referred to by the head managers was derived from the field of organizational theory [49]. One of the head managers reflected on this:

“Although I seem to have a lot of theoretical knowledge about implementation due to my higher education, this project has nevertheless taught me some new and more concrete tools on how to approach implementation.” (Hospital X, transcribed notes)

Both head managers used their knowledge from organizational theory in choosing implementation strategies, e.g., information on board meetings with all the staff and choosing early adopters with motivational arguments and tools. The burning platform was an example of a theoretical organizational tool, which is about understanding the importance of change through motivation. Although the head managers had learned about implementation through their education, they experienced that implementation science contained new knowledge and tools, which they considered to be useful in implementing the intervention.

#### Profession-related knowledge

A third form of theoretical justification for strategies emanated from professional knowledge, i.e., clinical knowledge concerning the treatment and care of patients. The analysis shows that clinically relevant knowledge related to different aspects of patient treatment and care depending on the profession. For the physicians, this knowledge could relate to diagnostics, for the physiotherapists it was related to respiratory physiotherapy, and for the nurses, administration of medications was discussed as clinically relevant knowledge. Particularly for the physicians and the physiotherapists, professional knowledge was mentioned as important in justifying why the intervention should be implemented. A physician explained:

“If I am going to motivate my medical colleagues to be active in implementing this intervention, they need the professional relevance. I need to have the clinical arguments ready.” (Hospital X, notes)

For this physician, justifications other than those pertaining to clinical professional knowledge seemed irrelevant. Accordingly, the physician chose the strategy of information at the physician conferences, because this was a forum for physicians to hear about clinically relevant topics and thus about the project and its implementation. Even though the research team had explained that information can rarely be used alone to change behavior, no other strategies were chosen.

For the physiotherapists, the justifications based on professional knowledge also increased motivation. An implementation champion expressed:

“An intervention with focus on mobility motivates physiotherapists. I cannot imagine that implementation is going to be a problem. I think many of our colleagues will require professional training to be sure they are up to date with the latest knowledge. It increases their motivation even more.” (Hospital Y, transcribed notes)

The implementation strategy of conducting ongoing training was selected by the physiotherapists based on a motivation to ascertain up-to-date clinically relevant knowledge rather than recognizing training as a means to improve skills. For physicians and physiotherapists, professional knowledge was used as a justification, but in different ways. For the physicians, it was a central way of getting their physician colleagues interested and motivated in the project. For the physiotherapists, motivation was strong from the beginning of the project, and was signaled through quotes such as: “*It is great to be allowed to work with the implementation of physical activity”* (Hospital X, notes) and: “*Physical activity is the core of our work”* (Hospital Y, notes). Their motivation was further strengthened by the strategy of conducting ongoing training*.*

In general, the group of frontline managers referred only to QI knowledge while head managers used theory from other scientific fields. Theoretical justification was used to select implementation strategies that could support and increase the motivation of the staff.

## Discussion

This qualitative study investigated the strategies selected by health care practitioners and their managers for the implementation of the WALK-Cph intervention. We found that implementation champions and managers selected primarily implementation strategies classified as educating and planning in the taxonomy by Powell et al. [13]. There were also a few managing quality, restructuring and financing strategies. The justifications for the selection of these strategies were made by using pragmatic justifications (experience, intentions, habits, resource limitations, sense of shared responsibility and unfamiliarity with implementation) and theoretical justifications (QI knowledge, organizational theory knowledge, and professional knowledge). These results are consistent with the taxonomy developed by Proctor et al. [3] concerning different types of justifications. We did not identify any empirical justifications to motivate selections of strategies.

The use of pragmatic justifications in our study highlights a tension between using generalizable research-based knowledge and paying attention to the importance of the local context [50]. Research has shown that clinical experts largely make choices based on a tacit practice-based knowledge built up from practitioners’ experience, which is manifested in their craft expertise and skills; the source is often specific problems that require solutions [51]. In contrast, some authors have suggested that implementation experts should make decisions based on findings from implementation research, i.e., research-based knowledge [3, 13]. However, there is a risk that research-based knowledge is difficult to apply in the local setting because it is not adapted to the specific context where implementation occurs, e.g., the type of leadership or culture in a particular hospital or department. On the other hand, a co-design process can produce more contextualized strategies and interventions, but the risk is that too much emphasis is put on pragmatic justifications of what clinical experts “believe” would work well because it may have worked in the past. It has been argued that unsuccessful implementation is more likely when strategies are chosen routinely or by habits rather than being based on purposefully addressing specific barriers [52].

Our study findings suggest the importance of finding a balance between research- and practice-based knowledge in co-design processes. Practice-based knowledge predominantly serves to solve the problems that occur in everyday life and work, but the subjective and context-bound nature of this knowledge limits its generalizability. On the other hand, research-based knowledge typically has ambitions for applicability beyond the immediate boundaries of the specific study, but this type of knowledge can rarely provide quick solutions to problems [52]. In practice, however, aspects of the two types of knowledge are intertwined, which means that it is rarely an either/or choice for health care practitioners, but more often a question of making sense of many sources of knowledge. Fitzgerald et al. [53] view the relationship as “circular”, with the two knowledge types reinforcing each other as they become woven together. An important point is that implementation is not only a science but also a practice since many implementers, as in this study, are not researchers. When implementation occurs in a real-world setting (and when not done by researchers) it requires judgment and skills to ensure adaptation to real-time changes and variations, as well as drawing on evidence from the field of implementation science. So, both types of knowledge are needed for practitioners to develop a high level of competence, i.e. the ability to act knowledgeably, effectively, deliberately, strategically and reflectively in a situation.

Many of the implementation strategies mapped onto the category of planning and educating [13], providing examples of selections based on practice-based evidence from past experiences. However, strategies like these tend to have a limited duration and may not support the implementation of an intervention throughout an entire project. The reason for choosing these short-term strategies may be due to the participants’ belief that implementation is an “introduction” of something new, i.e. implementation is something with a clear beginning and an end. However, if implementation is viewed in terms of a longer, more complex change process with no clear ending there is a need for strategies that can provide long-term support for implementation to be successful. Hence, the selection of strategies is dependent on the perspective of implementation. This finding highlights the relevance of selecting implementation strategies that can support the implementation process in a longer time perspective. One tool that could support the selection of strategies is for researchers to make a greater effort to relate these strategies to the participants and settings, so the participants become more familiar with the different types of implementation strategies and the underlying evidence. Evidence was not a factor that appeared to be significant to the participants in their justifications when no empirical justifications were used.

Education was a commonly selected category of implementation strategy by the stakeholders in our study. Despite the preference for this type of strategy there is rather weak evidence of effectiveness for educational strategies focusing on cognitive participation at the expense of collective action in changing healthcare professionals’ behaviours [54], which usually describes such strategies as ineffective for changing practice or achieving optimal care [44]. These results highlight the research-based knowledge versus practice-based knowledge dilemma, raising the question of whether a more research-led process would have ensured a stronger emphasis on strategies with proven effectiveness. Participatory design has seen a development from the users as subjects to users as partners, with a continuum of user-involvement methods in which the power to determine the outcome to a greater or lesser degree is placed with the researchers [22]. In the current study, the participants had full authority to influence the process and the outcome. Based on some of our results, including the choice of strategies being based only on pragmatic and theoretical justifications and the paucity of knowledge about implementation based on implementation research, a more equal distribution of power could possibly have been more appropriate. On the other hand, a more researcher-led process utilizing more research-based knowledge could challenge the basic premise of co-design, i.e., to ensure a high degree of user involvement and move decisional authority from the research team to the participants, also defined as “power from us to them” [55] and where the definition of power is the ability to influence an outcome [56].

The theoretical justifications in our study were not based on knowledge from implementation science [57]. This finding is not problematic per se, but the participants expressed a need to learn more about implementation research. Several of the participants were selected as implementation champions because they had previously dealt with QI tasks and implementation issues in their daily work. Our finding is consistent with Mosson et al. [58], who found that health care practitioners and their managers often lack skills for implementing evidence-based methods and that implementation often occurs without a structured approach [59].

These results suggest a need for expanding and/or improving training in implementation science for health care practitioners to facilitate the practical use of this knowledge. Westerlund et al. [60] and Lyon et al. [61] have highlighted a form of “implementation paradox,” wherein there is a risk of knowledge produced in implementation science not being used in real-world health care practice despite the fact that the field was borne out of ambitions to bridge the knowing-doing gap. Although implementation science is an applied science, the extent to which knowledge produced in this field is actually used by practitioners is not known [60]. There are few empirical studies concerning if or how knowledge on implementation is being applied in health care practice [62, 63]. Meissner et al. [38] and Ramaswamy et al. [64] argue that more courses on training in implementation science nationally and internationally are needed to expand implementation capacity [65]. Our experiences working with the implementation of the WALK-Cph intervention top managers need to acknowledge implementation research as a scientific field with relevant knowledge to support real-life implementation if they are to prioritize staff resources for learning about implementation science in the form of skills, knowledge, and practice-based learning.

A pragmatic justification that occurred in our study was a shared sense of responsibility, which can be understood as a relational concept where a person has responsibility for causing something to happen where there can be implications of praise or blame [66]. Achieving collaboration across health care sectors in Denmark is a complex matter [67–69], and the participants in our study had previously tried coordination of communications across health care sectors without much success. From earlier experiences, the participants had learned that there was not a one size fits all implementation strategy that could ensure efficient collaboration across sectors and a shared responsibility when implementing interventions. These previous experiences entailed developing a new strategy, a visit across sectors, which none of the participants had tried before. This was justified by the idea that it could create a shared responsibility, which would have consequences for the trust or distrust between the participants depending on the outcome of the implementation of the WALK-Cph intervention.

It has become increasingly important for managers to gain the trust of followers which is needed to achieve effective leadership [75]. Balkrishnan et al. [70] have defined trust as the willingness to be vulnerable to the actions of another party, irrespective of the ability to monitor or control the other party. To propose an implementation strategy that was not tried or known by some of the participants could be a sign of trust between the participants across sectors but also between the implementation champions and the managers. By creating a relational “safe” place built on trust in their interactions with the implementation champions, the managers made the participants more open to trying something new even if it meant failing [71]. Building trust became an important factor for pragmatic justifications.

### Strengths and limitations

A strength of the study was the design and methodological choices, which enabled us to follow the strategies from their selection to their implementation. This increased our knowledge about the “birth” and the “life” of implementation strategies in clinical practice. It was also a strength that we were present at the workshops to observe (gestures, mimics, etc.) the participants, which gave us a better understanding of the situational and contextual situation in which these strategies were selected. Another strength was that we described implementation strategies based on Proctor et al.’s [3] standards for characterizing implementation strategies, because this strengthened the labeling, increased comparability, and increased the knowledge of whether, which, who, and why when working with implementation strategies and health care practitioners.

A limitation of the study was that we could not comment on the effectiveness of the strategies selected. Further research is necessary to study whether there are correlations between the type of justification and management styles and effectiveness.

The way the co-design process was carried out may be considered a limitation. The researchers chose only to take on a productive role when contributing with implementation science knowledge and a part from this a facilitating role to ensure that ownership of the implementation plan would lie with the stakeholders. The ambition was to ensure that ownership of the implementation plan would lie with the stakeholders. Much emphasis was put on pragmatic justifications despite the fact that the research team had ensured that all relevant knowledge was present in the co-design team. Further, knowledge about implementation strategies was presented and discussed. Throughout the process, the facilitator asked challenging questions to the participants concerning their justifications of strategies.

The challenges faced in the co-design process may be difficult to generalize too broadly since many decisions taken were likely specific to the process and context of the studied case. Variability in co-design processes restricts generalizability and the ability to draw definitive or far-reaching conclusions.

A further limitation is the sole focus on health care practitioners’ training and learning implementation science instead of letting implementation researchers and health care practitioners train together as they could learn from each other, potentially making practice more research-informed and the science of implementation more practical and applicable.

A learning point for future co-design processes is to ascertain that expert knowledge and experience from the researchers, who were implementation researchers in this study, is afforded an equally central place in the co-design process. The goal would be to orchestrate the co-design process in a way that enables a synergistic combination of the different stakeholders’ expertise and experiences.

Transferability of the findings to other settings is possible, as thick descriptions were developed concerning the selection of implementation strategies and the justifications by selecting exemplary citations and describing the contexts of the data collection, including referring to the article focusing on reflective reflections on the use of co-design methods from a researcher perspective [36].

In this study, the implementation champions and managers came from medical departments and the municipalities. Further research should explore other settings and departments where implementation of evidence-based practice is more of a strategic imperative, which could yield a stronger focus on EBI. The study has shown that situational and relational factors are important for justification of implementation strategies, underscoring that justifications are highly sensitive to contextual factors such as culture, climate, and management.

## Conclusion

This qualitative study of implementation strategies selected by health care practitioners and their managers to support the implementation of an intervention to promote mobility found that implementation champions and managers predominantly selected implementation strategies that focus on planning for the implementation of the intervention and education by means of informing stakeholders about the intervention and/or its implementation. The selected strategies were motivated through pragmatic justifications, i.e. a clear rationale about what factors need to be addressed and how selected strategies may address them, and theoretical justifications based on theoretical knowledge accumulated in a research field. The sources of the theoretical knowledge were QI knowledge, organizational theory knowledge and professional knowledge. No implementation strategies were motivated by means of empirical justifications based on research evidence or previous experience with the evidence-based strategies. Although co-design processes may be very process- and context-specific, our finding points to a challenge in balancing strategies derived from practice-based knowledge and research-based knowledge.

## Supplementary Information

**Additional file 1: Appendix S1.** WALK-Cph intervention and implementation study, Hybrid II design.

**Additional file 2: Appendix S2.** Final WALK-Cph intervention.

**Additional file 3: Appendix S3.** Size and staff composition of Departments.

**Additional file 4: Appendix S4.** Selected implementation strategies.

**Additional file 5: Appendix S5.** Summary of the selected strategies and outcome effected.

## Data Availability

The datasets used and analysed during the current study are available from the corresponding author on reasonable request.

## Abbreviations

WALK-Cph: WALK-Copenhagen
EBI: Evidence-based intervention
