# Supplementary material for: Co-designing implementation strategies for the WALK-Cph intervention in Denmark aimed at increasing mobility in acutely hospitalized older patients: a qualitative analysis of selected strategies and their justifications
Source: BMC Health Serv Res. 2022 Jan 2;22:8. doi: 10.1186/s12913-021-07395-z (PMC8722331; doi:10.1186/s12913-021-07395-z)
Supplement: Supplementary file 1 — Additional file 1: Appendix S1. WALK-Cph intervention and implementation study, Hybrid II design. [file 12913_2021_7395_MOESM1_ESM.pptx]

## Slide 1
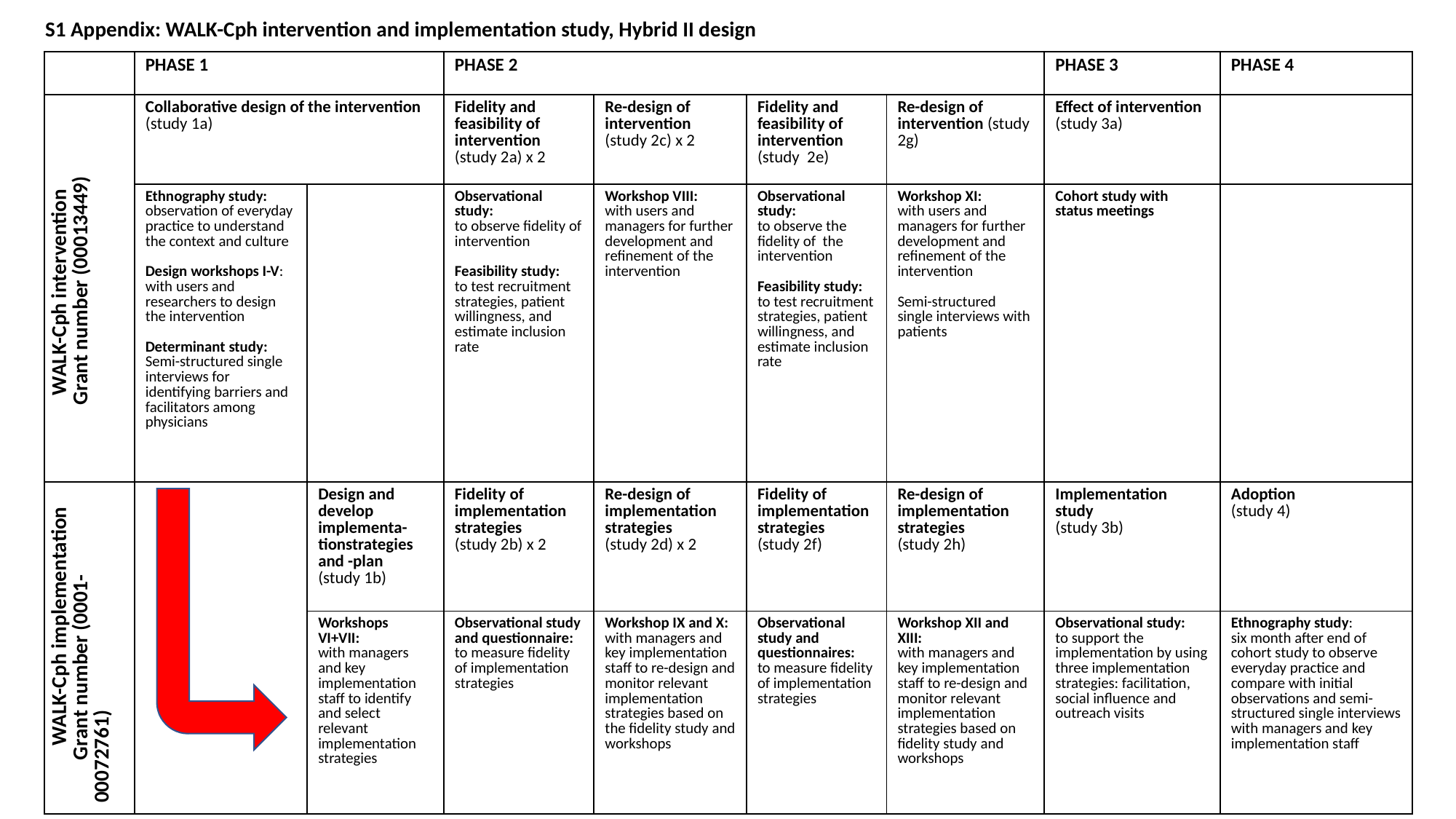

# S1 Appendix: WALK-Cph intervention and implementation study, Hybrid II design
| | PHASE 1 | | PHASE 2 | | | | PHASE 3 | PHASE 4 |
| --- | --- | --- | --- | --- | --- | --- | --- | --- |
| WALK-Cph intervention Grant number (00013449) | Collaborative design of the intervention (study 1a) | | Fidelity and feasibility of intervention (study 2a) x 2 | Re-design of intervention (study 2c) x 2 | Fidelity and feasibility of intervention (study 2e) | Re-design of intervention (study 2g) | Effect of intervention (study 3a) | |
| | Ethnography study: observation of everyday practice to understand the context and culture Design workshops I-V: with users and researchers to design the intervention Determinant study: Semi-structured single interviews for identifying barriers and facilitators among physicians | | Observational study: to observe fidelity of intervention Feasibility study: to test recruitment strategies, patient willingness, and estimate inclusion rate | Workshop VIII: with users and managers for further development and refinement of the intervention | Observational study: to observe the fidelity of the intervention Feasibility study: to test recruitment strategies, patient willingness, and estimate inclusion rate | Workshop XI: with users and managers for further development and refinement of the intervention Semi-structured single interviews with patients | Cohort study with status meetings | |
| WALK-Cph implementation Grant number (0001-00072761) | | Design and develop implementa-tionstrategies and -plan (study 1b) | Fidelity of implementation strategies (study 2b) x 2 | Re-design of implementation strategies (study 2d) x 2 | Fidelity of implementation strategies (study 2f) | Re-design of implementation strategies (study 2h) | Implementation study (study 3b) | Adoption (study 4) |
| | | Workshops VI+VII: with managers and key implementation staff to identify and select relevant implementation strategies | Observational study and questionnaire: to measure fidelity of implementation strategies | Workshop IX and X: with managers and key implementation staff to re-design and monitor relevant implementation strategies based on the fidelity study and workshops | Observational study and questionnaires: to measure fidelity of implementation strategies | Workshop XII and XIII: with managers and key implementation staff to re-design and monitor relevant implementation strategies based on fidelity study and workshops | Observational study: to support the implementation by using three implementation strategies: facilitation, social influence and outreach visits | Ethnography study: six month after end of cohort study to observe everyday practice and compare with initial observations and semi-structured single interviews with managers and key implementation staff |
