# Supplementary material for: Co-designing implementation strategies for the WALK-Cph intervention in Denmark aimed at increasing mobility in acutely hospitalized older patients: a qualitative analysis of selected strategies and their justifications
Source: BMC Health Serv Res. 2022 Jan 2;22:8. doi: 10.1186/s12913-021-07395-z (PMC8722331; doi:10.1186/s12913-021-07395-z)
Supplement: Supplementary file 2 — Additional file 2: Appendix S2. Final WALK-Cph intervention. [file 12913_2021_7395_MOESM2_ESM.docx]

**S2 Appendix: Final WALK-Cph intervention**

| COMPONENTS | INTERVENTION | DESIGN |
| --- | --- | --- |
| Welcome folder | On admission, health care professionals hand out a welcome folder, when introducing patients to the departments.  When handing out the welcome folder, health care professionals emphasize the importance of walking during and after hospitalization. | A WALK-Cph logo is printed on the front of the welcome folder.  The importance of walking during and after hospitalization is mentioned in the welcome folder. |
| Walk path | Daily, the patients are motivated by all health care professionals to use the walk path for walking and exercising.  The patients are introduced to the walk path and the poster exercises by the nurses, nursing assistants or the physiotherapists until the patients are self-reliant.  The patients should exercise by the walk path at least once a day | The WALK-project is assigned the colour green and a pair of green feet symbolizes the project. A walk path in the form of a line, is stuck on the floor, and marked with ’Walk path’ and green feet. The walking path is rectangular, and a rest area is located at each length of the rectangle and marked with a green chair and a poster with three exercises. Furthermore, a small whiteboard is placed by the rest area for the patients to mark the number of rounds or minutes they’ve walked. It is the responsibility of the night staff to update the board on a daily basis (count the number of lines on the whiteboard.) The following is written on the board:  Yesterday’s rounds/minutes  Today’s rounds/minutes |
| Posters with exercises placed in the wards | All health care professionals help motivate the patients to seek inspiration from the posters and perform the exercises by the pause areas of the walking path | The posters by the walking path are identical to the posters in the wards. The poster shows three exercises:   1. Walking: Walk by the walking path 2. Balance: Stand on one leg at a time (with a chair for aid) 3. Strength: Stand up from a chair and sit down |
| Physician prescribed walking plan | - Daily, the physiotherapists and the nurses cooperate on evaluating all of the ward’s patients to decide which patients to prescribe a walking plan, the level of the walking plan and possible changes to walking plan levels for those who have already been given a walking plan. The levels (colour) of all walking plans are noted on a patient board in the common office. - The physicians prescribe walking plans via the electronical patient jounal during rounds and motivate the patients to walk during the daytime. The physicians sign the walking plans before handing out to the patients - The walking plans are handed out to the patients by either the nurse or the physiotherapist - The walking plans are noted in the rehabilitation plans (GOP) by the physiotherapist (only if the patient is discharged with a GOP) - The walking plans are noted in the discharge reports by the nurse (if the patient has help at home). - All groups of professionals have the responsibility to motivate the patients to walk and follow the walking plans | Three different walking plans, with WALK logos, are preprinted to limit the amount of time required to hand out the walking plan:  Level 1 (red): 1 minute – three times a day  Level 2 (yellow): 5 minutes – three times a day  Level 3 (green): 10 minutes – three times a day  At level 3, three exercises are suggested on the walking plan as a supplement. The exercises are identical to the exercises on the posters.  The name of the patient must be written on the plan |
| Independent collection of clothes* | The patients collect clothes from the wardrobes themselves. On admission, the health care staff introduce the patients to the wardrobes and motivate/follow the patients to collect clothes. | The wardrobes, from which the patients may collect clothes, are marked with WALK logos |
| Independent collection of beverages | The patients collect beverages from the beverage wagon and the refrigerator.  On admission, the health care staff introduce the patients to the beverage wagon and the refrigerator and motivate the patients/follow the patients to collect beverages |  |
| After discharge, patients with a walking plan, who are discharged with a rehabilitation plan, will be contacted by phone by a municipal therapist | At discharge, the physiotherapist makes sure to note in the rehabilitation plan that the patient is discharged with a walking plan.  1-5 days after discharge, a therapist from the municipality contacts the patient by phone to motivate the patient to continue to follow the walking plan |  |
| After discharge, patients with a walking plan, who are discharged without a rehabilitation plan but receive home care, will be contacted by phone by home health care personnel | At discharge, the nurse makes sure to note in the discharge papers for the municipality that the patient is discharged with a walking plan.  After discharge, when visiting the patient, the home care health care personnel motivate the patient to follow their walking plan |  |

*This component was not a part of the intervention in Department Y
