# Supplementary material for: Co-designing implementation strategies for the WALK-Cph intervention in Denmark aimed at increasing mobility in acutely hospitalized older patients: a qualitative analysis of selected strategies and their justifications
Source: BMC Health Serv Res. 2022 Jan 2;22:8. doi: 10.1186/s12913-021-07395-z (PMC8722331; doi:10.1186/s12913-021-07395-z)
Supplement: Supplementary file 3 — Additional file 3: Appendix S3. Size and staff composition of Departments. [file 12913_2021_7395_MOESM3_ESM.docx]

**S3 Appendix: Size and staff composition of Departments**

|  | **Beds** | **Staff** | **Nurses** | **Nursing assistants** | **Physicians** | **Therapists** |
| --- | --- | --- | --- | --- | --- | --- |
| Department of Endocrinology (X) | n=24 | n=36 | n=18 | n=6 | n=12 |  |
| Department of General Medicine (Y) | n=25 | n=37 | n=18 | n=6 | n=8 | n=5* |

* At Department Y, the physiotherapists are affiliated to the department
