# Supplementary material for: Co-designing implementation strategies for the WALK-Cph intervention in Denmark aimed at increasing mobility in acutely hospitalized older patients: a qualitative analysis of selected strategies and their justifications
Source: BMC Health Serv Res. 2022 Jan 2;22:8. doi: 10.1186/s12913-021-07395-z (PMC8722331; doi:10.1186/s12913-021-07395-z)
Supplement: Supplementary file 4 — Additional file 4: Appendix S4. Selected implementation strategies. [file 12913_2021_7395_MOESM4_ESM.docx]

**S4 Appendix: Selected implementation strategies**

**A) Implementation strategies selected from the Department of Endocrinology at Hospital X**

| **Name of the strategy** | **Definition of the strategy** | **The actors** | **The action** | **The targets** | **Temporality** | **Dose** | **Implementation outcome affected** | **Justification** |
| --- | --- | --- | --- | --- | --- | --- | --- | --- |
| Change physical structure and equipment | Adapt the physical structure and equipment to best accommodate the targeted innovation | The frontline managers at the Endocrinological Department and the architect | In the Department of Endocrinology, a walk-path is attached to the floor in the form of stickers and posters with exercises are put on the walls of the department | The staff’s opportunity to provide the intervention | Before the beginning of testing the intervention.  **(**QIF-phase 1) | One week to plan and develop the physical changes | Feasibility | Pragmatic evidence from the frontline managers |
| Conduct local consensus discussions [1,2, 4,5,6] | Include stakeholders in discussions that address whether the chosen problem is important and whether the clinical intervention to address it is appropriate | The frontline managers and the implementation champions | Managers meet with local stakeholders and discuss clarifications on how, precisely, the intervention should proceed. Ongoing follow-up discussions on the intervention and how it is progressing. | An opportunity to involve and motivate the staff and secure ownership of the intervention and implementation | Weekly meetings  (QIF-phase 1,2 and 3) | Meetings of 10 to 30 minutes | Acceptability and fidelity | Pragmatic evidence from the frontline managers |
| Develop educational materials [1] | Develop written material in person and by email | The frontline managers and the quality coordinator in the department | Written material about the intervention is placed on boards in the department and distributed in writing in electronical newsletters | Securing dissemination of knowledge about the intervention | Before the beginning of testing the intervention.  (QIF – phase 1) | Develop the material to hang on boards and writing in the newsletter once a month | Fidelity and adoption | Pragmatic evidence from the frontline manager |
| Information on board meetings with all the staff [1,2, 4, 5] | Inform about the intervention and its progress | The physicians who are responsible for the implementation and the frontline managers | Staff participating in the board meeting get information about the intervention and an update about the process | Increase knowledge on an individual level and motivation on a collective level | Information at board meetings (QIF - phase 1,2 and 3) | Twice a week | Adoption | Pragmatic evidence from the physicians and frontline managers |
| Information at physician conferences [4,5] | Inform about the intervention and its progress | The physicians responsible for implementation | Physicians participating at the conferences get information about the intervention and an update about the process | Increase knowledge on an individual level and motivation on a collective level | Information at conferences  (QIF - phase 1,2 and 3) | Weekly | Acceptability | Pragmatic evidence from the physicians |
| Tailor strategies [1,2,3,4,5,6, 7, 8, 9,10] | Tailor the implementation strategies to address barriers and facilitators that were identified through earlier data collections | The frontline managers and the implementation champions | As part of the implementation workshops, implementation strategies are discussed and developed to address the identified barriers | Minimize resistance to the intervention and the implementation at a collective level (staff) | Before starting the WALK-project and along the way in the project (QIF – all phases) | Four × 1 hour | Acceptability and fidelity | Pragmatic evidence from the group |

*The numbers in brackets show which barriers are associated with the strategy.

QIF: Qualitative Implementation Framework. Phases; 1) Initial considerations regarding the host setting, 2) Creating a structure for implementation, 3) Ongoing structure once implementation begins and 4) Improvement of future applications.

**B) Implementation strategies selected from the Department of Occupational- and Physical Therapy at Hospital X**

| **Name of the strategy** | **Define the strategy** | **The actors** | **The action** | **The targets** | **Temporality** | **Dose** | **Implementation outcome affected** | **Justification** |
| --- | --- | --- | --- | --- | --- | --- | --- | --- |
| Change physical structure and equipment | Adapt the physical structure and equipment to best accommodate the targeted innovation | The frontline managers at the Endocrinological Department and the architect | In the Endocrinological Department, a walk-path is attached to the floor in the form of stickers and posters with exercises are put on the walls of the department | The staff’s opportunity to provide the intervention | Before the beginning of testing the intervention.  **(**QIF-phase 1) | One week to plan and develop the physical change | Feasibility | Pragmatic evidence from the head - and frontline managers |
| Conduct local consensus discussions [3] | Include stakeholders in discussions that address whether the chosen problem is important and whether the clinical intervention to address it is appropriate | The head and the frontline managers | Managers meet with local stakeholders and discuss clarifications on how the intervention should proceed and ongoing follow-up discussions on the intervention and how it is progressing. | An opportunity to involve and motivate the staff and secure ownership of the intervention | Weekly meetings  (QIF- phase 1,2 and 3) | Meeting of 10 to 30 minutes | Acceptability and fidelity | Pragmatic evidence from the head managers |
| Identity of early adopters [3,7] | Identify early adopters at the local site to learn from their experiences with implementing interventions in daily practice | The head and frontline managers | The head and frontline managers identify and talk about their experiences with implementation of interventions with focus on increasing the mobility of patients | Secure that all relevant daily practice experiences are heard at a collective level and involve and motivate the staff | Before starting the WALK-project  (QIF – phase 1) | One hour | Acceptability and adoption | Pragmatic evidence from the head - and frontline managers |
| Mandate change [8] | Declaring the priority of the intervention and their determination to have it implemented | The head and frontline mangers | At various meetings inform and ask about the intervention, follow up, listen to the therapists and their experiences with the intervention and the process. | Secure collective motivation and progress of the implementation process | Weekly meetings  (QIF - phase 1,2,3) | Meetings of 10 to 30 minutes | Acceptability and adoption | Theoretical evidence from the head managers. Pragmatic evidence from the frontline managers |
| Conduct ongoing training [3] | Plan for and conduct training in the clinical intervention ongoingly | The development management physiotherapist | All new employees learn about the walk intervention as part of their introductory program | Increase knowledge and skills at an individual level | Once a month (QIF – all phases) | 30 minutes | Feasibility and fidelity | Theoretical evidence from the head – and frontline managers |
| Tailor strategies  [1,2,3,4,5,6, 7, 8, 9,10] | Tailor the implementation strategies to address barriers and facilitators that were identified through earlier data collections | The head and frontline managers | As part of the implementation workshops, implementation strategies are discussed and developed to address the identified barriers | Minimizing resistance against the intervention and the implementation at a collective level (staff) | Before starting the WALK-project and along the way in the project (QIF – all phases) | Four x 1 hour | Acceptability and fidelity | Pragmatic evidence from the group |

*The numbers in brackets show which barriers are associated with the strategy.

**C) Implementation strategies selected from the implementation champions at the rehabilitation departments in Municipality X**

| **Name of the strategy** | **Define the strategy** | **The actors** | **The action** | **The targets** | **Temporality** | **Dose** | **Implementation outcome affected** | **Justification** |
| --- | --- | --- | --- | --- | --- | --- | --- | --- |
| Conduct local consensus discussions [3] * | Include central stakeholders in discussions that address whether the chosen problem is important and whether the clinical intervention to address it is appropriate | Head managers and the implementation champions | Managers meet with local stakeholders and discuss clarifications on how, precisely, the intervention should y proceed. Ongoing follow-up discussions on the intervention and how it’s progression. | An opportunity to involve and motivate the staff and secure ownership of the intervention | Weekly meetings  (QIF - phase 1,2 and 3) | Meetings of 10 to 30 minutes | Acceptability and fidelity | Pragmatic evidence from the head managers and the implementation champions. |
| Mandate change [8] | Declaring the priority of the intervention and their determination to have it implemented | The head managers | At various meetings inform and ask about the intervention, follow up, listen to the therapists and their experiences with the intervention and the process. | Secure collective motivation and progress in the implementation process | Weekly meetings  (QIF - phase 1,2,3) | Meeting of 10 to 30 minutes | Acceptability and adoption | Theoretical and pragmatic evidence from the head managers and Pragmatic evidence from the implementation champions |
| Conduct ongoing training [3] | Plan for and conduct training in the clinical intervention ongoingly | The development physiotherapists | All new employees learn about the walk intervention as part of their introductory program | Increase knowledge and skills at an individual level | Once a month (QIF – all phases) | 30 minutes | Feasibility and fidelity | Theoretical and pragmatic evidence from both the head manager and the implementation champions. |
| Information at board meetings with all staff [3] | Inform about the intervention and its progress | The implementation champions | Staff participating in the board meeting get information about the intervention and an update about the process | Increase knowledge at an individual level and motivation on a collective level | Twice a week (QIF – all phases) | 10-15 minutes | Acceptability and adoption | pragmatic evidence from implementation champions |
| Develop educational materials [3] | Develop a guideline that makes it easier for staff to deliver the intervention | The implementation champions | Staff that contact the citizens at home follow the guidelines to secure that the right questions about the intervention are asked | Secure that the fidelity of the intervention is increased | Every time the staff contact the citizens (QIF – all phases) | 5-10 minutes | Fidelity | Pragmatic evidence from the implementation champions |
| Reminder [3] | Develop a reminder system designed to help staff to remember the intervention | The implementation champions | In the citizens’ rehabilitation plans, a star will indicate that the citizen haves a walk-plan | Secure that the staff remember to ask about the walk-plan when talking with the citizens | One time before the intervention is started  (QIF – phase 1) | A week | Acceptability and fidelity | Pragmatic evidence from the implementation champions |
| Build a coalition | Cultivate relationships with all the partners in the projects | The head managers from the rehabilitation department, and from the occupational and physical therapy department as well as the frontline manager from the Endocrinological Department | Cross-sectorial visit between the rehabilitation department and the Endocrinological Department | Secure that all partners have knowledge about work processes in each other's departments and how the intervention affects and interrelates with the different departments. | One time before the intervention is started  (QIF – phase 1) | Three hours | Adoption | Pragmatical evidence from the head managers |
| Tailor strategies [1,2,3,4,5,6, 7, 8, 9,10] | Tailor the implementation strategies to address barriers and facilitators that were identified through earlier data collections | The head manager and the implementation champions | As part of the implementation workshops, implementation strategies are discussed and developed to address the identified barriers | Minimizing resistance against the intervention and the implementation at a collective level (staff) | Before starting the WALK-project and along the way in the project period (QIF – all phases) | Four x 1 hour | Acceptability and fidelity | Theoretical evidence from the head managers. |
| Audit and feedback [3] | Monitor how many citizens have received a walk-plan when discharged from the hospital | The implementation champions | Monitor walk-plans | Continued feedback will motivate to perform the intervention | Once a month (QIF – phases 2,3 and 4) | 30 minutes | Fidelity and adoption | Pragmatic evidence from the head manager and the implementation champions |

*The numbers in brackets show which barriers are associated with the strategy.

**D) Implementation strategies selected from the implementation champions at the Rehabilitation Department in Municipality Y**

| **Name of the strategy** | **Define the strategy** | **The actors** | **The action** | **The targets** | **Temporality** | **Dose** | **Implementation outcome affected** | **Justification** |
| --- | --- | --- | --- | --- | --- | --- | --- | --- |
| Conduct local consensus discussions [3] * | Include central stakeholders in discussions that address whether the chosen problem is important and whether the clinical intervention to address it is appropriate | The implementation champions | Managers meet with colleagues and discuss clarifications on how, precisely, the intervention should proceed and ongoing follow-up discussions on the intervention and how it is progressing. | An opportunity to involve and motivate colleagues and secure ownership of the intervention | Weekly meetings  (QIF - phase 1,2 and 3) | Meeting of 10 to 30 minutes | Acceptability and fidelity | Pragmatic evidence from the implementation champions. |
| Reminder [3] | Development of a schedule to measure citizens, who have been prescribed a walk-plan. | The implementation champions | The implementation champions ask colleagues about the usability of the schedule to measure the number of walk-plans | Measurement of the number of walk-plans could motivate the staff to remember to ask the citizens about the walk-plans | Weekly (QIF – phase 1) | 10 minutes | Fidelity | Pragmatic evidence from the implementation champions |
| Information meeting with head managers | Meeting with the head manager | The implementation champions | Status meetings to update the managers | To secure management support | Before starting the WALK-project and along the way in the project period (QIF – all phases) | 15 minutes | Acceptability | Pragmatic evidence from the implementation champions |

*The numbers in brackets show which barriers are associated with the strategy.

**E) Implementation strategies selected from the implementation champions at the Department of General Medicine at Hospital Y**

| **Name of the strategy** | | **Define the strategy** | | **The actors** | | **The action** | | **The targets** | | **Temporality** | | **Dose** | | **Implementation outcome affected** | | **Justification** |
| --- | --- | --- | --- | --- | --- | --- | --- | --- | --- | --- | --- | --- | --- | --- | --- | --- |
| Conduct local consensus discussions [3] * | | Include central stakeholders in discussions that address whether the chosen problem is important and whether the clinical intervention to address it is appropriate | | Frontline managers and the implementation champions | | Managers meet with local stakeholders and discuss clarifications on how, precisely, the intervention should proceed. Ongoing follow-up discussions on the intervention and how it is progressing. | | An opportunity to involve and motivate the staff and secure ownership of the intervention | | Weekly meetings  (QIF - phase 1,2 and 3) | | Meetings of 10 to 30 minutes | | Acceptability and fidelity | | Pragmatic evidence from the frontline managers and the implementation champions. |
| Information on board meetings with all staff [3] | | Status about the intervention and its progress | | The implementation champions | | Staff participating in the board meeting get information about the intervention and an update about the process | | Increase knowledge at an individual level and motivation on a collective level | | Every day (QIF – all phases) | | 10-15 minutes | | Acceptability and adoption | | Pragmatic evidence from implementation champions |
| Audit and feedback [3] | | Monitor how many patients receive a walk-plan in the hospital | | The implementation champions | | Monitor walk-plans | | Continued feedback will motivate to perform the intervention | | Every day (QIF – phases 2,3 and 4) | | 10 minutes | | Fidelity and adoption | | Theoretical and pragmatic evidence from the frontline manager and the implementation champions |
| Develop educational materials [1] | Develop written material and smart phrases | | The implementation champions | | Written material about the importance of mobility is posted in the department | | Securing dissemination of knowledge about the intervention | | Before the beginning of testing the intervention.  (QIF – phase 1) | | Develop the material | | Fidelity and adoption | | Pragmatic evidence from the implementation champions | |
| Conduct ongoing training [3] | | Plan for and conduct training in the clinical intervention ongoingly | | The development management physiotherapist | | All new employees learn about the walk intervention as part of their introductory program | | Increase knowledge and skills at an individual level | | Once a month (QIF – all phases) | | 30 minutes | | Feasibility and fidelity | | Theoretical and pragmatic evidence from both the frontline managers and the implementation champions. |

*The numbers in brackets show which barriers are associated with the strategy.
