# Supplementary material for: Co-designing implementation strategies for the WALK-Cph intervention in Denmark aimed at increasing mobility in acutely hospitalized older patients: a qualitative analysis of selected strategies and their justifications
Source: BMC Health Serv Res. 2022 Jan 2;22:8. doi: 10.1186/s12913-021-07395-z (PMC8722331; doi:10.1186/s12913-021-07395-z)
Supplement: Supplementary file 5 — Additional file 5: Appendix S5. Summary of the selected strategies and outcome effected. [file 12913_2021_7395_MOESM5_ESM.docx]

**S5 Appendix: Summary of the selected strategies and Outcome affected**

With regard to **the actor,** the responsibility for executing the strategies were head managers (9 strategies), frontline managers (10 strategies), implementation champions (15 strategies) and implementation physicians (2 strategies). The design architect, the quality coordinator and the development physiotherapists were expected to execute the remaining three strategies.

The **action targets** were predominantly at individual and collective levels. Only one strategy was targeted at the organizational level (*Build a coalition)*. No strategy was targeted at an institutional level.

Concerning **temporality**, 5 of the strategies were expected to be executed before testing of the intervention in QIF phase 1 (*Change physical structure and equipment, Identity early adopters, Reminder,* *Build a coalition and Develop educational materials).* Three of the strategies were expected to be executed in QIF phases 1,2 and 3 (*Conduct local consensus discussions, Information at the physician conferences and Mandate change*). One strategy was expected to be executed in QIF phases 2,3 and 4 (*Audit* *and feedback*). Five of the strategies were expected to be executed in all QIF phases (*Tailor strategies, Conduct ongoing training,* *Information on board meetings with all the staff,* *Develop educational materials and Information meeting with head managers).* One strategy, *Develop educational materials,* was selected both as a strategy to be executed in the beginning of the process, when the materials were developed, and to continue through all QIF phases in which the material was hung on boards and written in an electronic newsletter.

The **dose** of the strategies was from 10 minutes to one week. Short dosages were on strategies that were carried out daily, e.g. board meetings, while longer dosages were initial and as major follow-ups.

**Outcome affected:** Two of the selected strategies (*Change physical structure and equipment and Conduct ongoing training*) would affect the feasibility of the implementation. Eight strategies were expected to affect the acceptability of the implementation *(Conduct local consensus discussions, Information at the physician conferences, Tailor strategies, Identity early adopters, Mandate change, Information on board meetings with all the staff, Reminder and Information meeting with head managers).* Fidelity was expected to be affected by six implementation strategies *(Conduct local consensus discussions,* *Develop educational materials, Tailor strategies, Conduct ongoing training, Reminder, Audit and feedback).* Similarly, adoption was expected to be affected by six strategies *(Develop educational materials, Information on board meetings with all the staff, Identity early adopters, Mandate change, Build a coalition,* *Audit and feedback).*
